# Gust Response and Alleviation of Avian-Inspired In-Plane Folding Wings

**DOI:** 10.3390/biomimetics9100641

**Published:** 2024-10-18

**Authors:** Haibo Zhang, Haolin Yang, Yongjian Yang, Chen Song, Chao Yang

**Affiliations:** 1 School of Aeronautic Science and Engineering, Beihang University, Beijing 100191, China; zhanghaibo@buaa.edu.cn (H.Z.); yanghaolin@buaa.edu.cn (H.Y.); yangyj1@aeroht.com (Y.Y.); yangchao@buaa.edu.cn (C.Y.); 2 Institute of Systems Engineering, China Academy of Engineering Physics, Mianyang 621900, China; 3 Guangdong Huitian Aerospace Technology Co., Guangzhou 511400, China

**Keywords:** bioinspired aircraft, folding wing, aeroelasticty, gust response, viscous vortex particle

## Abstract

The in-plane folding wing is one of the important research directions in the field of morphing or bionic aircraft, showing the unique application value of enhancing aircraft maneuverability and gust resistance. This article provides a structural realization of an in-plane folding wing and an aeroelasticity modeling method for the folding process of the wing. By approximating the change in structural properties in each time step, a method for calculating the structural transient response expressed in recursive form is obtained. On this basis, an aeroelasticity model of the wing is developed by coupling with the aerodynamic model using the unsteady panel/viscous vortex particle hybrid method. A wind-tunnel test is implemented to demonstrate the controllable morphing capability of the wing under aerodynamic loads and to validate the reliability of the wing loads predicted by the method in this paper. The results of the gust simulation show that the gust scale has a significant effect on the response of both the open- and closed-loop systems. When the gust alleviation controller is enabled, the peak bending moment at the wing root can be reduced by 5.5%∼47.3% according to different gust scales.

## 1. Introduction

For decades, aeronautic engineers have continued to draw inspiration from the flight of birds, resulting in a variety of morphing aircraft design concepts and control mechanisms [1,2,3]. Researchers have observed that various birds change the folding angles of their wings due to flight states switching [4]. Modern research showed that this ability gives birds excellent maneuverability and gust-resistance, which are usually better than similarly sized unmanned aerial vehicles (UAVs). This discovery has spurred the emergence and subsequent development of in-plane folding wings. However, very little work has discussed how to quantify or analyze the ability of these wings to resist gust disturbances. One of the most critical problems in this field is to accurately calculate the loads on the wing during its folding process, which is usually measured in experiments in the past.

Grant et al. [5] designed what may be the first bird-shaped UAV with in-plane folding mechanisms, using a tract and runner system to keep the artificial feathers together. This defines the basic form of this type of wing. The current research on in-plane folding wings has primarily focused on the structural realization of the folding function. Specifically, the design schemes are primarily centered around reproducing the wrist-joint control mechanism of bird wings [6,7,8].

Although extensive research has been conducted on the structural realization of in-plane folding wings, there are still many challenges in evaluating their performance. To quantitatively study the aerodynamic characteristics of avian-inspired wings, the most common research method is wind-tunnel testing. The results of some wind-tunnel tests have verified that the folding mechanism of the wing can benefit in terms of delaying stall [9], reducing drag [10,11], or conducting maneuvers [12]. During the morphing process, the trailing edge of the wing is folded inward through an overlapping mechanism, which typically relies on fan-shaped thin-plate structures or authentic bird feathers. To maintain the smoothness of the wing’s surface, this overlapping structure is often designed to be relatively thin, resulting in a significant reduction in structural stiffness at the feather region compared to the main wing segment. The aerodynamic loads on the wing will also vary due to structural elastic deformation [10], and the same applied to bird feathers [11]. Moreover, Roshanbin et al. [13] have also found that wing flexibility has a positive effect on bionic aircraft by enhancing lift force, reducing mechanical power consumption, and decreasing noise levels. However, there is currently no research work that explores the interaction between the in-plane folding wing structure and aerodynamic forces. Therefore, it is still difficult to accurately assess the specific performance benefits offered by folding wings.

In order to understand and quantitatively assess the control effect of in-plane folding of the wing on gust loads, the first problem that must be addressed is the aeroelasticity modeling of the wing. There are numerous theoretical problems in the aeroelasticity modeling of in-plane folding wings. These challenges originate from two main sources: the transient response analysis of time-varying structures and the unsteady aerodynamic modeling in the folding process. In similar studies, time-varying effects due to structural morphing are usually ignored.

In recent years, some scholars have used Hamilton’s law for general dynamical systems to study the transient response of the systems containing time-varying parameters and obtained simulation results comparable to the analytical solutions on some simple models [14,15,16,17]. This idea may help to advance the modeling and research of large-scale morphing vehicles. In addition, the viscous vortex particle model has made progress in the simulation of aerodynamic loads on complex wakes [18,19], bionic UAVs [20], and the new conceptual aircraft [21] due to its meshless characteristic. These results provide a basis for aeroelasticity modeling of new conceptual morphing wings, including the in-plane folding wing.

The core contribution of this paper is to establish a time-domain aeroelasticity model that can take into account the time-varying effects of wing folding, which can provide a theoretical basis for aerodynamic load analysis and gust simulation of elastic in-plane folding wings. By approximating the change of structural properties in each time step, a method for calculating the structural transient response expressed in recursive form is obtained. On this basis, an aeroelasticity model of the wing is developed by coupling with the aerodynamic model using the unsteady panel/viscous vortex particle hybrid method. We have implemented a wind-tunnel test to verify the effectiveness of the aeroelasticity model in simulating wing loads during the folding process. All the above methods and models give the theoretical basis for the simulation of the gust response of in-plane folding wings and the design of gust alleviation controllers. Subsequently, we build a gust model and study the gust response and alleviation problems of the wing. The potential ability of the in-plane folding to alleviate gust loads is verified by numerical simulation, which is related to the gust scale.

## 2. Structural Design and Prototype Manufacturing

The first key issue in the study on in-plane folding wings or similar avian-inspired wings is the design and realization of the folding mechanism and wing structure. Birds have flexible and widely distributed nerve-muscle intelligent control and actuation systems, making it an almost unattainable challenge to replicate the control mechanism of bird wings. Up to now, there is no artificial sensor or actuator of the efficiency exceeding that of biological systems.

**Figure 1:**
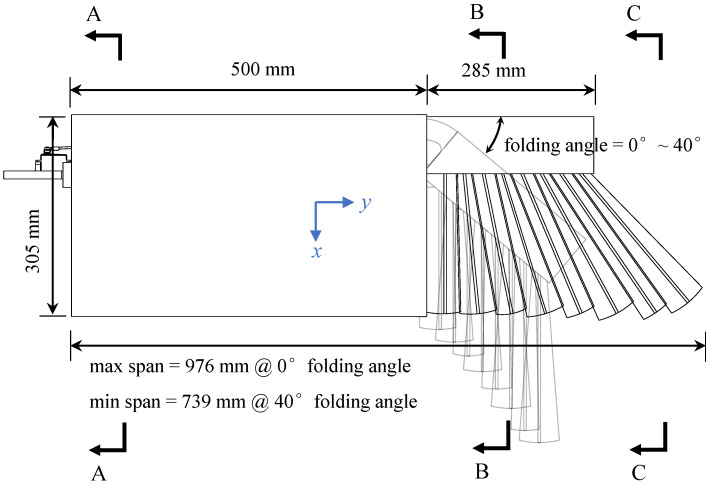
Scheme of the in-plane folding wing.

**Figure 2:**
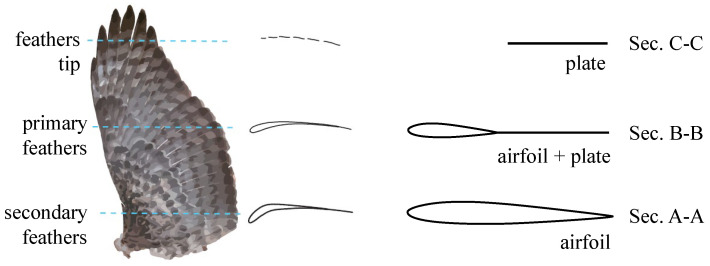
Cross-section shape of the wing.

We have made some necessary simplifications by extracting the core mechanism of bird wing shape control. This helps to make the final design scheme easy to implement. As shown in Figure 1, we present an in-plane folding wing based on an artificial feather mechanism. The outer segment of the wing is made of fan-shaped plates to simulate the in-plane folding function of the primary feathers of bird wings [22]. The cross-section shape of each wing segment is compared to that of a bird wing in Figure 2. The cross-section markings in Figure 2 correspond to those in Figure 1.

The servo system is fixed at the wing root and connected to the foldable section by a linkage mechanism. The outermost feather is fixedly connected to the leading edge, while other feathers are elastically connected to each other by rubber ropes.

**Figure 3:**
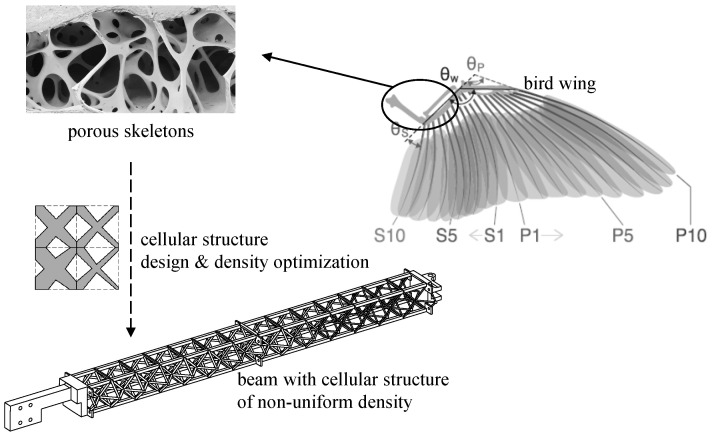
Design of skeleton-inspired beam.

In addition to the morphing function, bird bones are usually constructed from porous structured materials and, thus, are considered to be lightweight and of high specific strength. A similar design, the cellular structure, is used to build the main beam of the wing, therefore improving its overall mechanical properties. The design of the skeleton-inspired beam with cellular structure is shown in Figure 3.

In this section, we will first present the structural design of the main beam and its optimization methodology. On this basis, more details of the folding mechanism and the manufacturing of the wing prototype will be demonstrated.

### 2.1. Beam with Cellular Structure of Non-Uniform Density

The skeletons of birds are not solid structures but consist of porous structures. This structural feature is an evolution result to adapt the high energy expenditure in flight, helping to increase the specific strength of skeletons and to achieve lightweight.

The topology of the cells in a cellular structure significantly influences the macroscopic mechanical properties of the structure. The regular octahedral lattice with a high coordination number is a commonly used construction unit and has been used to build lightweight morphing wings in recent years [23,24,25]. In our design framework, the beam consists of two rows of octahedral cells, where the cross-section areas of the bars in each octahedral cell are considered to be independent design variables. By allocating the relative densities of the different units and configuring the material to the appropriate spatial location, the utilization efficiency of the material can be improved, helping to indirectly reduce the weight of the structure.

In order to solve the optimal design problem of the beam through standard optimization algorithms, it is abstracted into the following mathematical model:

(1) $$\left\{\begin{array}{cc} \mathrm{minimize}:\; & c\left(x\right)=u_{\mathrm{tip},L}^{2}+u_{\mathrm{tip},T}^{2}+p\sum_{i}x_{i}^{2},\\ \mathrm{subject}\;\mathrm{to}:\; & \left\{\;\begin{array}{cc} \; & \mathrm{Ku}=f,\\ \; & x_{\min}\leq x_{i}\leq x_{\max},\\ \; & \frac{u_{\mathrm{tip},L}-u_{\mathrm{tip},T}}{L_{c}}\leq \tan\theta_{\max}, \end{array}\right. \end{array}\right.$$

where x is the vector of design variables, representing the ratio of the cross-section areas of the bars in each octahedral cell to the reference value. Moreover, $u_{\mathrm{tip},L}$ and $u_{\mathrm{tip},L}$ represent the displacement of the wing tip at the leading and trailing edge, respectively. The objective function c(x) consists of two parts: the square of wing tip displacement and the sum of squares of $x_{i}$. This means that the optimization model can consider the overall performance of the structure in terms of stiffness and weight. In the constraints, K is the stiffness matrix of the structure, u is the vector of node displacements, and f is the vector of node loads. In addition, $L_{c}$ is the chord length of the wing, and $\theta_{\max}$ is the upper limit of the permissible torsion angle.

The load f is obtained by calculating the aerodynamic loads of the wing at a fully expanded state under typical working conditions. The stiffness matrix K is obtained by rotating the sub-stiffness matrices of each beam and superimposing them. The Euler-Bernoulli beams can be used to model the beam system within the cellular structure since the shear stresses in the octahedral cells are weak under usual working conditions [23].

To solve the optimization problem (1), the available algorithms include traditional gradient-based algorithms and heuristic algorithms. In this paper, the gradient-based algorithm is used to solve this model. However, note that the objective function c(x) contains the displacement term *u*, but the derivative of the displacement *u* with respect to structural parameter x is difficult to compute. The adjoint sensitivity method will help to compute the gradient information required by the gradient-based solver. We construct the augmented Lagrange function:

(2) $$\bar{c}=c+\lambda^{T}\left(f_{F}-K_{\mathrm{FF}}u_{F}\right),$$

where $\lambda^{T}$ is the Lagrange multiplier, also known as the adjoint vector. The subscript F denotes the part of the matrix or vector that corresponds to free nodes. Considering the balanced equation, we can know that $\bar{c}=c$ always holds. According to the chain rule, the full derivative of Equation (2) is

(3) $$\frac{dc}{dx_{i}}=\frac{d\bar{c}}{dx_{i}}=\left(\frac{𝜕c}{𝜕u_{F}}-\lambda^{T}K_{\mathrm{FF}}\right)\frac{du_{F}}{dx_{i}}+\frac{𝜕c}{𝜕x_{i}}+\lambda^{T}\left(\frac{df_{F}}{dx_{i}}-\frac{dK_{\mathrm{FF}}}{dx_{i}}u_{F}\right).$$

Considering that $\bar{c}=c$ holds true for any value of $\lambda^{T}$, here the particular $\lambda^{T}$ is chosen to satisfy

(4) $$\frac{𝜕c}{𝜕u_{F}}-\lambda^{T}K_{\mathrm{FF}}\equiv 0.$$

Thus,

(5) $$\lambda^{T}=\frac{𝜕c}{𝜕u_{F}}K_{\mathrm{FF}}^{-1}=\frac{𝜕}{𝜕u_{F}}\left(u_{\mathrm{tip},L}^{2}+u_{\mathrm{tip},T}^{2}\right)K_{\mathrm{FF}}^{-1}.$$

Substituting it back into Equation (3), the gradient of the objective function can be obtained:

(6) $$\frac{dc}{dx_{i}}=\frac{𝜕c}{𝜕x_{i}}+\lambda^{T}\left(\frac{df_{F}}{dx_{i}}-\frac{dK_{\mathrm{FF}}}{dx_{i}}u_{F}\right)=2px_{i}-\lambda^{T}\frac{dK_{\mathrm{FF}}}{dx_{i}}u_{F}.$$

Equation (6) can be easily calculated through the structural model.

However, even if the design sensitivity in model (1) is calculated, there will be another challenge to find its solution. This is due to the large number of components in the design variable x and the significant difference in the magnitude of design sensitivity relative to each component. To compute this model with sparse parameters, an optimization algorithm known as AdaDelta [25] is used. The core of the algorithm lies in the addition of a relaxation factor to the update rate, which is related to the historical gradient information in the iterative process:

(7) $$x_{i}^{t+1}=x_{i}^{t}-\frac{\eta_{0}}{\sqrt{E{\left[g_{i}^{2}\right]}^{t}+\varepsilon }}\cdot g_{i}^{t},$$

where $\eta_{0}$ is the initial update rate that is specified, and $g_{i}=dc/dx_{i}$. The superscript *t* represents the number of the iteration step, and the subscript *i* represents the number of the design variable component. The relaxation factor in Equation (7) contains an infinitesimal value ε to prevent numerical singularity, and

(8) $$E{\left[g_{i}^{2}\right]}^{t}=\rho \cdot E{\left[g_{i}^{2}\right]}^{t-1}+\left(1-\rho \right)\cdot {\left[g_{i}^{2}\right]}^{t},$$

where ρ is the weighting factor for cumulative values of the square of design sensitivity. Using the result given by Equation (6) and the iterative algorithm given by Equation (7), the optimization model (1) can be solved.

**Figure 4:**
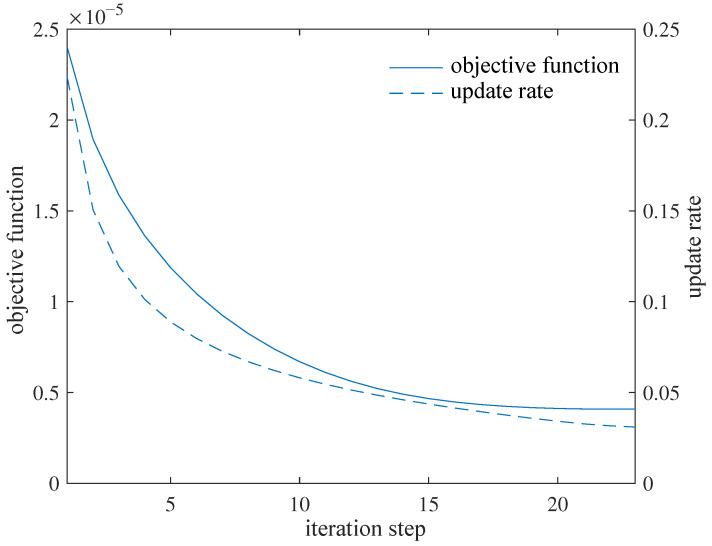
Iteration process for optimization design.

The beam designed in this paper is 3D-printed with (heat-treated) aluminum alloy of the elasticity modulus E=60±10 GPa. In this example, a conservative valuation of E=50 GPa is used. The rods within each octahedral cell have circular cross-sections with a radius of $r_{i}=r_{0}x_{i}$, where the reference radius is $r_{0}=1.0$ mm. The typical flight conditions are set as angle of attack $\alpha =5^{\circ }$ and airspeed v=15 m·s${\,}^{-1}$. Referring to the process limitations of 3D printing using aluminum alloys, the minimum cross-section radius can be set as $x_{\min}=0.7$. The maximum permissible torsion angle under the typical condition is $\theta_{\max}=5^{\circ }$. The initial value $x_{i}=2.0$, the initial update rate $\eta_{0}=0.05$, the penalty coefficient for structure weight $p=2\times 10^{-7}$, and the weighting factor ρ=0.95 are chosen in the optimizer. The optimization model (1) is solved under the above setup conditions, and the iteration process is shown in Figure 4, where the update rate is defined as $\max|x_{i}^{t+1}-x_{i}^{t}|$ for every time step *t*.

**Figure 5:**
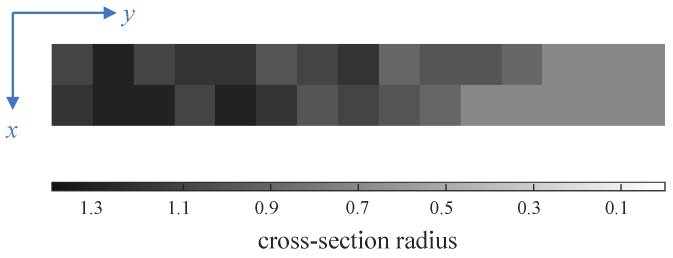
Optimized result of cross-section radius distribution of rods in cells.

**Figure 6:**
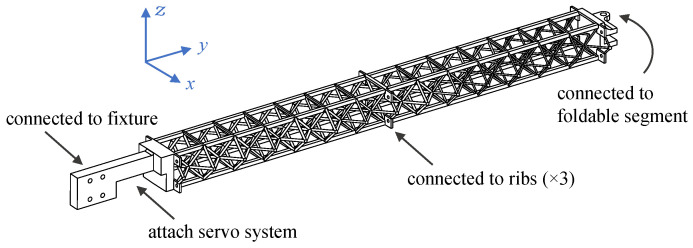
Beam with cellular structure of non-uniform density.

As shown in Figure 4, as the iteration process proceeds, the optimizer adaptively increases and decreases the update rate of design variables, resulting in a fast convergence to a good result shown in Figure 5. The 3D reconstruction according to the optimized result and further detailed design leads to the beam shown in Figure 6.

### 2.2. Detailed Design of Folding Mechanisms and Connectors

**Figure 7:**
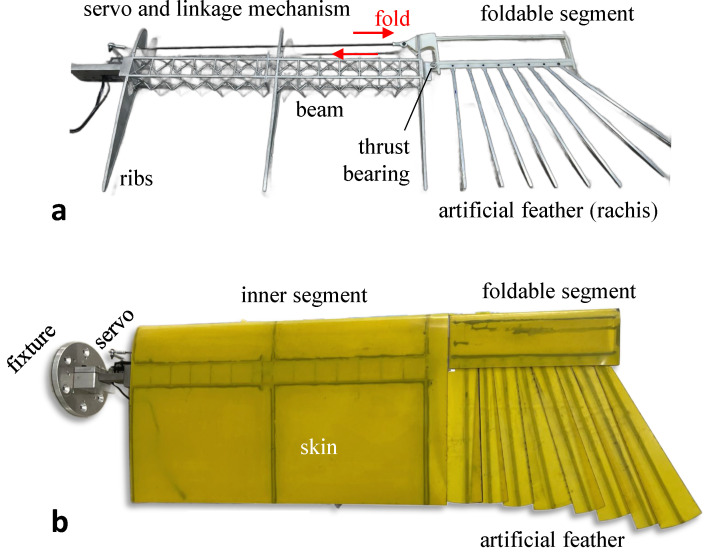
Prototype of avian-inspired in-plane folding wing. (**a**) Skeleton only. (**b**) Skin is attached.

The foldable segment of the wing is connected to the outer side of the beam through a thrust bearing, as Figure 7a. The servo is connected to the leading edge of the foldable segment through a linkage mechanism and controls the linkage to be pushed out or retracted, thus driving the folding or unfolding of the foldable segment. Except for the outermost artificial feather that is locked and connected to the foldable segment, the other feathers and the foldable segment are connected by miniature bearings and are elastically connected to each other by rubber ropes, as Figure 7b, so that the whole feather area can be expanded or folded just like a folding fan. The process and material programs not mentioned above can be checked in Table 1.

The weight of the wing prototype is 1007 g, excluding the weight of the servo. The prototype allows programmable control of the folding angle between $0^{\circ }$∼$40^{\circ }$ with a single servo. The projected area of the wing can be increased by up to 22.3% (0.219 m${\,}^{2}$ to 0.269 m${\,}^{2}$), and the wingspan can be increased by up to 32.1% (739 mm to 976 mm) when the wing is fully expanded. This prototype provides the basis for the subsequent modeling and wind-tunnel test of in-plane folding wings.

## 3. Aerodynamic Loads of Elastic Wings

Birds often fly in complex natural wind environments at low speeds (typical Mach number range is between 0.01 to 0.08 [26]) and are susceptible to gust disturbances. By actively folding their wings in gusts, birds can gain the ability to mitigate the influence of gust disturbances. This effect arises mainly from the change in the effective wing area and, therefore, the aerodynamic load control.

Although the gust response is also associated with the change in body attitude, it is currently difficult to model this process completely now due to the effect of fluid-structure coupling and the change in body inertial [4,27]. A large number of relevant studies in the past have mainly used research methods based on observation or experimentation. In this paper, we will first start from the aeroelasticity modeling, and try to simulate the aerodynamic loads of elastic in-plane folding wings from the perspective of physical theory.

### 3.1. Aeroelasticity Modeling

Since the outer segment of an in-plane folding wing usually consists of artificial feathers made up of a single layer of skin, its stiffness is significantly reduced compared to that of the inner segment. It leads to a deviation between the aerodynamic loads of the elastic wing and the rigid model through the unloading effect [10]. In order to simulate the aerodynamic loads of the elastic wing in an airflow (and/or a gust), it is necessary to first develop an aeroelasticity model of the wing.

There are two main difficulties in aeroelasticity modeling: transient response analysis of time-varying structures and unsteady aerodynamic modeling during the folding process. During the folding process of the wing, the relative positions of each component are constantly changing, resulting in the time-varying mass and stiffness distributions. In addition, the accurate wake evolution is also difficult to be simulated as the in-plane folding of the wing may cause spatially interfere between the motion range and the wake region of the wing.

#### 3.1.1. Structure Modeling

The structural dynamic model of the wing is established through a spatio-temporal discretization of Hamilton’s law for general dynamical systems [14,15]. Hamilton’s law states that the trajectory of any real physical system meets the condition that makes its Lagrange function action S(q) to take an extreme value:

(9) δS(q)=0,

where

(10) $$S\left(q\right)=\int_{t_{1}}^{t_{2}}L\left(q,\dot{q},t\right)dt.$$

The vector q is the generalized coordinate of the system. The following transformation relationship between the generalized coordinate $q_{j}$ and the physical coordinates $r_{i}$ (of discretized nodes) is often used in analytical mechanics:

(11) $$\left\{\begin{array}{cc} \frac{𝜕{\dot{r}}_{i}}{𝜕{\dot{q}}_{j}}= & \frac{𝜕r_{i}}{𝜕q_{j}},\\ \frac{𝜕{\dot{r}}_{i}}{𝜕q_{j}}= & \frac{d}{dt}\left(\frac{𝜕r_{i}}{𝜕q_{j}}\right). \end{array}\right.$$

According to D’Alembert’s principle, the virtual work done by the combined force (including the inertial forces and binding forces) is equal to zero:

(12) $$\delta W=\sum_{i=1}^{N}\left(m_{i}{\ddot{r}}_{i}+{\dot{m}}_{i}{\dot{r}}_{i}-f_{i}-{\dot{m}}_{i}v_{0i}\right)\delta r_{i}=0,$$

where $v_{0i}$ is the velocity of the added/removed mass relative to the inertial reference system. Based on this relationship and transforming it into the generalized coordinate representation, an expression of Hamilton’s law can be obtained:

(13) $$\int_{t_{0}}^{t_{f}}\left(\delta L+\sum_{j}\left(Q_{j}^{\mathrm{nc}}+R_{j}+Z_{j}\right)\delta q_{j}\right)dt-\sum_{j}\frac{𝜕T}{𝜕{\dot{q}}_{j}}\delta q_{j}{|}_{t_{0}}^{t_{f}}=0,$$

where

(14a) $$\begin{array}{cc} Q_{j}^{\mathrm{nc}} & =\sum_{i=1}^{N}f_{i}^{\mathrm{nc}}\frac{𝜕r_{i}}{𝜕q_{j}}, \end{array}$$

(14b) $$\begin{array}{cc} R_{j} & =\sum_{i=1}^{N}{\dot{m}}_{i}v_{0i}\frac{𝜕r_{i}}{𝜕q_{j}}, \end{array}$$

(14c) $$\begin{array}{cc} Z_{j} & =\sum_{i=1}^{N}\left(\frac{1}{2}\frac{d}{dt}\left({\dot{r}}_{i}^{2}\frac{𝜕m_{i}}{𝜕{\dot{q}}_{j}}\right)-\frac{1}{2}{\dot{r}}_{i}^{2}\frac{𝜕m_{i}}{𝜕q_{j}}\right). \end{array}$$

The superscript nc denotes physical quantities associated with non-conservative forces, and *T* is the kinetic energy. In addition to the conventional generalized force $Q_{j}$ associated with the nodal loads, the Equation (13) adds additional generalized forces $R_{j}$ and $Z_{j}$ associated with the mass change, as well as a process-independent non-integral residual term. This is the main difference between time-varying and time-invariant systems.

**Figure 8:**
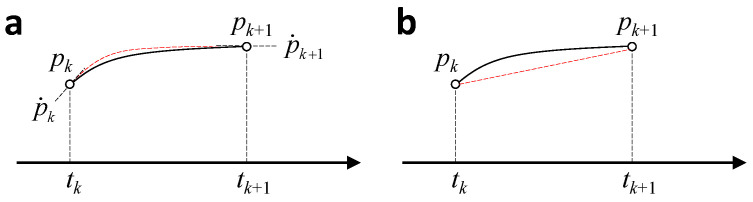
Parameter approximating within a single time step. (**a**) Hermite interpolation for structural parameters. (**b**) Linear interpolation for generalized coordinates.

Equation (13) gives the continuous time form of Hamilton’s law, but for numerical simulation, it also needs to be discretized into time steps. Hermite and linear interpolation were used to approximate the changes in structural parameters and generalized coordinates within each time step, respectively, as shown in Figure 8.

By approximating the parameters and integrating the coefficients of the formula at each time step, Hamilton’s law expressed in recursive form can be given:

(15) $$H_{1}\left[\begin{array}{c} q_{k}\\ \Delta t{\dot{q}}_{k} \end{array}\right]=H_{0}\left[\begin{array}{c} q_{k-1}\\ \Delta t{\dot{q}}_{k-1} \end{array}\right]+H_{f}\left[\begin{array}{c} {\bar{f}}_{k-1}\\ \Delta t{\dot{\bar{f}}}_{k-1}\\ {\bar{f}}_{k}\\ \Delta t{\dot{\bar{f}}}_{k} \end{array}\right],$$

where the coefficient matrices $H_{1},H_{0},H_{f}$ all depend on the specific treatments of the non-integral residual term in Equation (13). Equation (15) is also named time finite element formulations (TFEM). The coefficient matrices used in this paper can be found in Appendix A. According to Equation (15), the transient structural response of the wing during the folding process can be computed numerically.

#### 3.1.2. Aerodynamic Modeling

In addition to the difficulties in structural response, unsteady aerodynamic modeling of in-plane folding wings is also a major difficulty. In this paper, the traditional unsteady panel method (UPM) is improved by introducing the discrete viscous vortex model to avoid mesh distortion or numerical singularity problems caused by mesh extrusion in the wake region.

The discrete viscous vortex model is an approach to simulate low-speed viscous flows using viscous vortex particles with vector vorticity, which has shown potential in complex wake simulation [18] or bionic aerodynamics [20] in recent years. Thus, it is also known as the viscous vortex particles method (VVPM). The core of VVPM is the discretization of the velocity-vorticity form of the Navier–Stokes equations for uncompressible flows in three dimensions [19]:

(16) $$\frac{d}{dt}\omega =\omega \cdot \nabla u+\nu \nabla^{2}\omega ,$$

where u is the flow velocity, ν is the kinematic viscosity coefficient. ∇ is the gradient operator: $\nabla ={\left[\frac{𝜕}{𝜕x},\frac{𝜕}{𝜕y},\frac{𝜕}{𝜕z}\right]}^{T}$, $\nabla^{2}$ is the Laplace operator: $\nabla^{2}={\left[\frac{{𝜕}^{2}}{𝜕x^{2}},\frac{{𝜕}^{2}}{𝜕y^{2}},\frac{{𝜕}^{2}}{𝜕z^{2}}\right]}^{T}$, and ω=∇×u is the vorticity. By discretizing the real vorticity field, the vorticity distribution at the specific location x can be described by vortex particles:

(17) $$\omega \left(x,t\right)=\sum_{i}\xi_{\sigma }\left(x-x_{i}\right)\alpha_{i},$$

where $x_{i}$ and $\alpha_{i}$ are the position vector and vorticity vector of the *i*-th vortex particle at moment *t*, respectively. $\xi_{\sigma }\left(\cdot \right)$ is the Gaussian smoothing distribution function containing the smoothing parameter σ:

(18) $$\xi_{\sigma }\left(\rho \right)=\frac{1}{\sigma^{3}}\xi \left(\rho \right)=\frac{1}{{\left(2\pi \right)}^{3/2}\sigma^{3}}e^{-\rho^{2}/2}.$$

Therefore, the flow field of Equation (16) can be described using vortex particles:

(19a) $$\begin{array}{cc} \; & \frac{dx_{i}}{dt}=u_{i}, \end{array}$$

(19b) $$\begin{array}{cc} \; & \frac{d\alpha_{i}}{dt}=\alpha_{i}\cdot \nabla u_{i}+\nu \nabla^{2}\alpha_{i}. \end{array}$$

According to the Helmholtz decomposition, the velocity field u of an uncompressible flow field can be decomposed into an irrotational velocity field and a rotational velocity field. When the flow field is modeled by the UPM/VVPM hybrid method, the rotational velocity field can be induced only by vortex particles. The specific algorithm for this induced velocity field is

(20) $$u_{\omega }\left(x,t\right)=\sum_{i}K\left(x-x_{i}\right)\times \alpha_{i}.$$

The Rosenhead–Moore approximation is used for the kernel function K(·):

(21) $$K\left(x-x_{i}\right)=\frac{1}{4\pi }\frac{x-x_{i}}{(|x-x_{i}{{|}^{2}+R_{v}^{2})}^{3/2}},$$

where $R_{v}$ is the cut-off parameter.

The first term on the right-hand side of Equation (19b) is calculated using the direct derivation of the velocity field:

(22) $$\alpha_{i}\cdot \nabla u\left(x_{i},t\right)=\sum_{j}\frac{1}{\sigma^{3}}\left[\alpha_{j}\times \right]\cdot \nabla \left[K\left(x_{i}-x_{j}\right)\right]\cdot \alpha_{i},$$

where $\left[\alpha_{j}\times \right]$ is the skew-symmetric matrix expression the vorticity vector $\alpha_{j}$.

However, the spatial derivatives of the parameters are difficult to compute since VVPM uses a meshless modeling approach. The particle strength exchange method (PSE) [28] is therefore used to calculate the second term on the right-hand side of Equation (19b), whose core is to approximate the Laplace operator through an integral function. Thus,

(23) $$\nu \nabla^{2}\alpha_{i}=\frac{2\nu }{\sigma^{2}}\sum_{j}\xi_{\sigma }\left(\right|x_{i}-x_{j}\left|\right)\left(V_{i}\alpha_{j}-V_{j}\alpha_{i}\right).$$

The value of the above equation decays very quickly with increasing distance between vortex particles, so it is sufficient to consider only the other vortex particles in the vicinity of each particle in the actual calculation.

**Figure 9:**
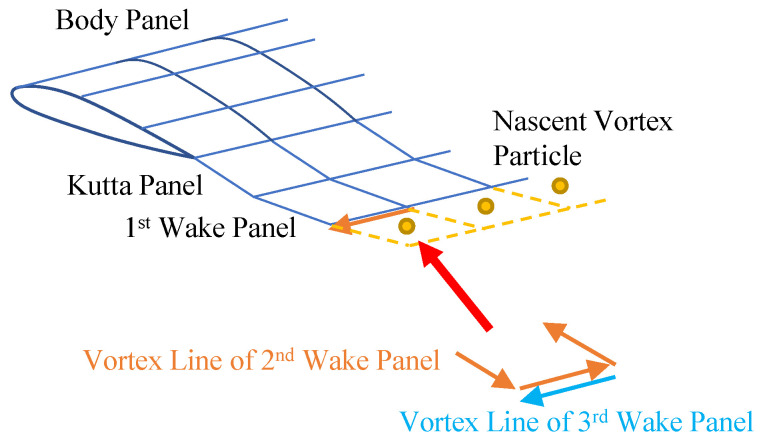
Unsteady panel/viscous vortex particle hybrid method.

Equation (19) determines the law of vortex particle evolution in the flow field. The vorticity of the nascent vortex particles at each moment is calculated by the standard UPM, as Figure 9. At the beginning moment of each time step, the first row of the wake panel is generated by the standard UPM and will be transformed into equivalent vortex particles [29] at the beginning moment of the next time step.

It should be noted in particular that since the flow field induced by vortex particles cannot be described by a scalar potential function, this portion of the induced velocity will be merged into the motion velocity of the wing surface for compatibility with the potential function calculation of the standard UPM. This treatment is easy to implement and will not be discussed in detail here.

#### 3.1.3. Coupling

**Figure 10:**
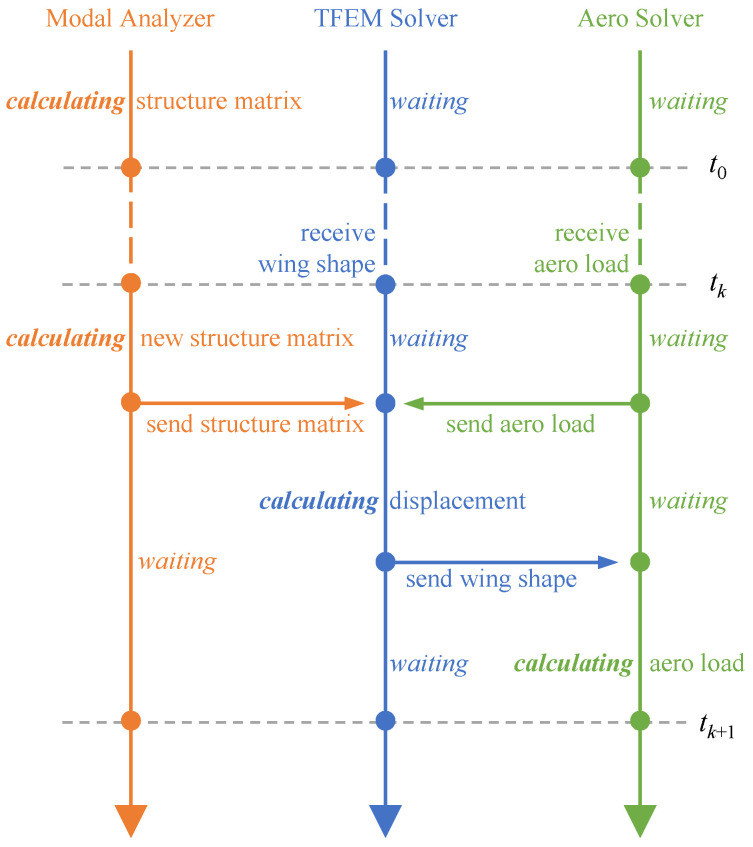
Coupling workflows of aeroelasticity model of in-plane folding wings.

To realize the simulation of flow around the elastic wing during its in-plane folding process, we developed a framework that can be used for the fluid-structure coupled simulation of time-varying structure, as Figure 10.

At the beginning of each time step, the modal analyzer first calculates the current structure matrices (including the modal matrix) based on the new folding angle and sends them to the TFEM solver through the coupling interface. At the same time, the aerodynamic load solver also sends the aerodynamic loads calculated at the end of the previous time step to the TFEM solver. Therefore, the TFEM solver can calculate the new position of the structure after this time step according to Equation (15). The new wing shape is then recorded and sent to the aerodynamic load solver through the coupling interface. Finally, the aerodynamic load solver calculates the aerodynamic loads at the end of this time step using the new wing shape and records them. According to the above coupling workflows, the time-domain fluid-structure coupling simulation during the wing folding process can be achieved using three solvers alternately.

As the complex character of structural damping, it is difficult to obtain the structural damping matrix theoretically. The damping of the foldable structure has been simplified by adopting a fixed modal damping ratio for each mode. These values are obtained through a ground modal test and presented in Table 2. If further consideration of the damping ratios at different folding angles is required, Equation (15) and Equations (A1)–(A3) can be utilized to calculate the structure response after a relationship between the damping ratio and the folding angle is established.

### 3.2. Numerical Simulation and Wind-Tunnel Test

**Figure 11:**
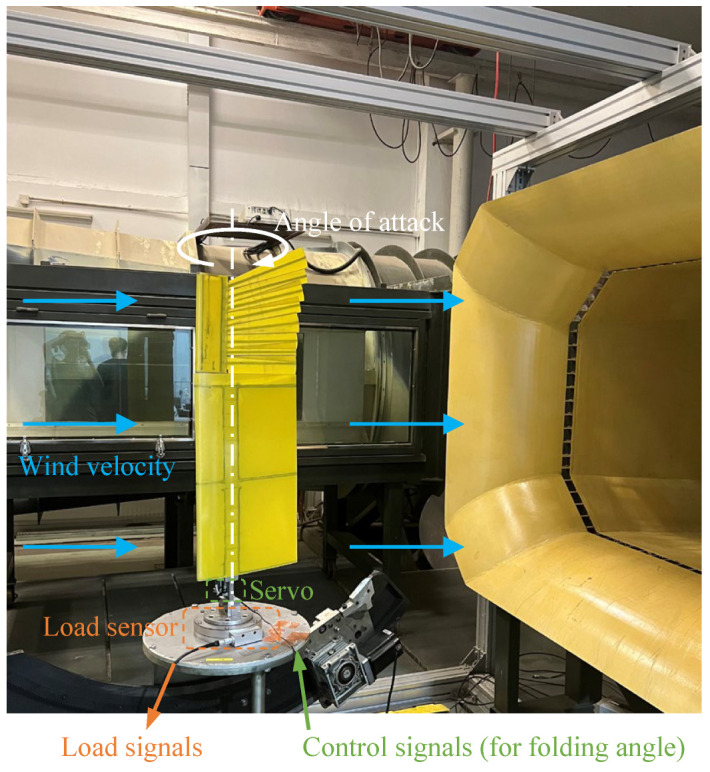
Wind-tunnel test of the wing prototype.

A wind-tunnel test of the wing prototype is performed to validate the wing loads predicted by the aeroelasticity model. The prototype is mounted vertically in the wind tunnel to avoid interference from gravity loads, as Figure 11. The wing prototype is connected to the flange of the six-component load sensor via the fixture and then attached vertically to the platform within the wind tunnel through a chassis, enabling the angle of attack of the wing to be controlled by the sideslip angle controlling mechanism inside the wind tunnel. During the wind-tunnel test, the folding angle of the wing is set to keep at a certain angle (known as steady working condition) or vary according to a fixed frequency sinusoidal law (known as unsteady working condition), which is achieved by the servo at the wing root. This servo is connected to a computer via a PC-based multi-function I/O device (with a digital-to-analog converter, not shown in Figure 11), allowing the computer to send commands to control the folding angle of the wing.

**Figure 12:**
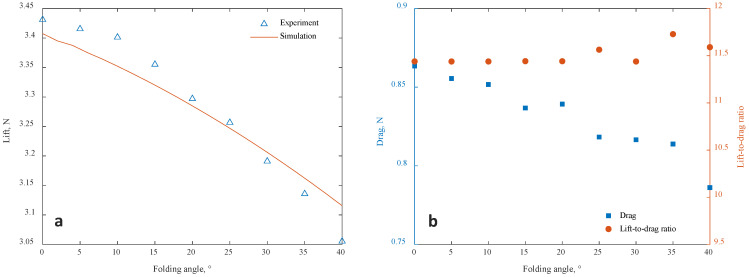
Comparison of the wing loads at the steady working condition (u=10 ms${\,}^{-1}$, $\alpha =5^{\circ }$). (**a**) Lift. (**b**) Drag and lift-to-drag ratio.

**Figure 13:**
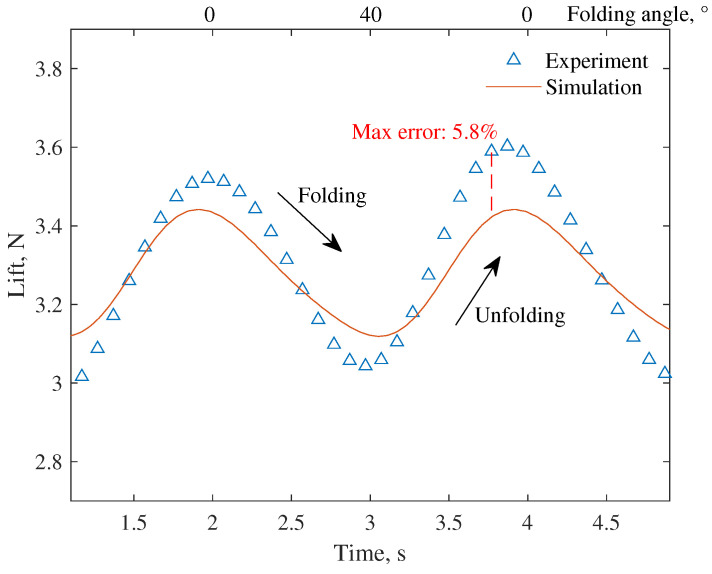
Comparison of the wing lift results at the unsteady working condition (u=10 ms${\,}^{-1}$, $\alpha =5^{\circ }$, f=0.5 Hz).

The comparison of the wing lift tested in the wind-tunnel test and the numerical simulation results obtained from the aeroelasticity model is shown in Figure 12a and Figure 13, where *u* is the wind velocity, α is the angle of attack, and *f* is the folding frequency. Under typical working conditions, the method in this paper can predict and track the lift change well. The max simulation errors are 2.0% and 5.8% for steady case and unsteady case, respectively. It should be noted that there is an asymmetry in the lift change during the folding and unfolding process, which is caused by the additional velocity of the wing surface motion. It may also be related to the different characteristics of the vortex system development during folding and unfolding [10]. This feature will lead to the hysteresis of the lift, thus invalidating the static model. A more detailed study of this mechanism awaits further development in flow display experiments or high-resolution computational fluid dynamics (CFD) studies.

The drag measured under different folding angles in the wind-tunnel test is presented in Figure 12b. This result has not been directly compared with the simulation outcomes from the theoretical model, as the aerodynamic model proposed in this paper is incapable of simulating the frictional drag of the wing. This discrepancy leads to underestimation of the drag in the theoretical model. If a precise theoretical calculation of drag values is required, a high-fidelity CFD model should be employed. Nevertheless, since the gust load of the wing is primarily influenced by lift, the utilization of the algorithm presented in this paper does not affect subsequent gust-related research.

According to the results of the steady working condition, both the lift and drag of the wings are correlated with the folding angle, and the variation in the lift-to-drag ratio of the wing with respect to the folding angle is not significant. When the wings on both sides are folded asymmetrically, the aircraft can obtain a rolling moment due to the lift difference between the two sides. However, the drag difference will also introduce additional yawing moments at the same time. This indicates that wing folding cannot be employed to control the aircraft’s attitude in a decoupled manner. The rudder is an optional joint control surface that can be utilized to compensate for the additional yawing effect resulting from asymmetric wing folding.

## 4. Gust Response and Alleviation

In the following section we will introduce a simple gust model and combine it with the aeroelasticity model to demonstrate the response characteristics of the in-plane folding wing in vertical gusts and the potential application in gust alleviation.

### 4.1. Gust Modeling

Actual gusts in nature are complex, but in order to establish a theoretical model of gust perturbation and response, the gusts can be simplified based on the extraction of the main features of them. In this paper, the disturbance velocity approach (DVA) is used to model the gusts, which allows the flux balance to be changed by superimposing the disturbance velocity on the original aerodynamic model to simulate the gust disturbance field [30,31]. In subsequent case studies and discussions, it is assumed that the gust field acts in the vertical direction, which represents the most severe condition in gust response problems. If different gust angles or wing angles of attack need to be considered, only the direction of the gust velocity or the incoming flow velocity needs to be adjusted accordingly without requiring any additional modifications to the model itself.

We use a standard “1 − cos” gust model. Its computational model can be represented by the following equation:

(24) $$u_{G}=\left\{\begin{array}{ccc} \; & \frac{u_{\mathrm{ds}}}{2}\left[1-\cos\left(\frac{\pi s}{H}\right)\right],\; & 0\leq x\leq 2H,\\ \; & 0,\; & \mathrm{otherwise}, \end{array}\right.$$

where $u_{G}$ is the gust velocity, $u_{\mathrm{ds}}$ is the peak gust velocity, *s* is the travelling distance into the gust area, and H=2λ is the gust scale. Equation (24) simulated a gust area fixed to the ground. In reference to commonly used design specifications, in the standard “1 − cos” gust model, the peak gust velocity should be considered in structural design and can be determined according to the gust scale:

(25) $$u_{\mathrm{ds}}=u_{\mathrm{ref}}F_{g}{\left(\frac{H}{350}\right)}^{1/6},$$

where the reference gust velocity $u_{\mathrm{ref}}$ and the flight profile mitigation factor $F_{g}$ are both related to the flight altitude. Considering the low altitude range of most of the bionic UAVs, the effect of flight altitude is ignored in this paper, and all velocities are taken as the equivalent velocities at sea level. The flight profile mitigation factor $F_{g}$ is set as 1, which is on the conservative side.

**Figure 14:**
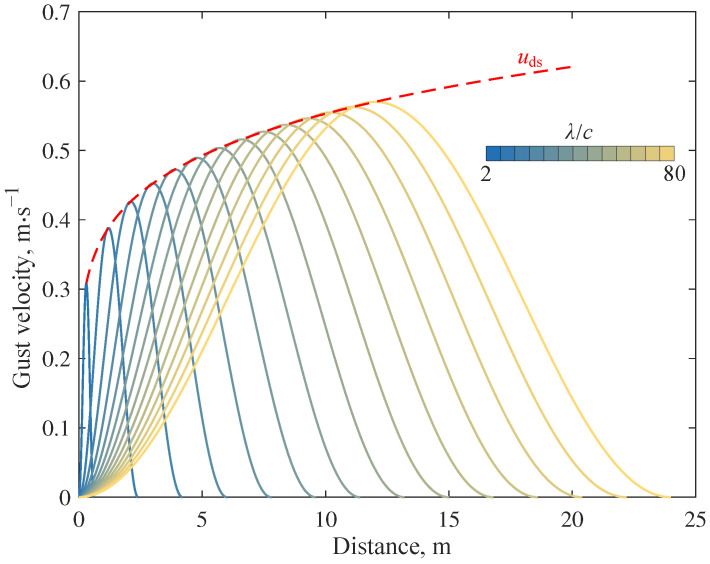
Gust field velocity curve.

Taking the reference gust velocity $u_{\mathrm{ref}}=1$ ms${\,}^{-1}$ and the gust scale λ=2c∼80c (*c* is the chord length of the wing), the gust velocity field at different scales can be obtained as shown in Figure 14.

### 4.2. Gust Response

**Figure 15:**
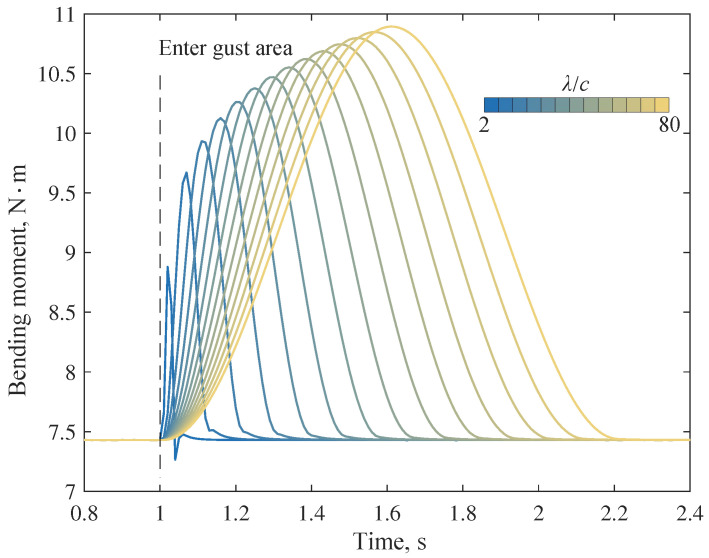
Bending moment at the wing root.

Under the flight condition of u=20 ms${\,}^{-1}$ and $\alpha =5^{\circ }$, it is assumed that the wing enters the gust area at t=1 s and the wing root is fixed. Before the wing enters the gust area, the folding angle $\theta =0^{\circ }$. Then, the bending moment response at the wing root is shown in Figure 15. The bending moment of this example is calculated from the internal forces in the structural finite element model.

**Figure 16:**
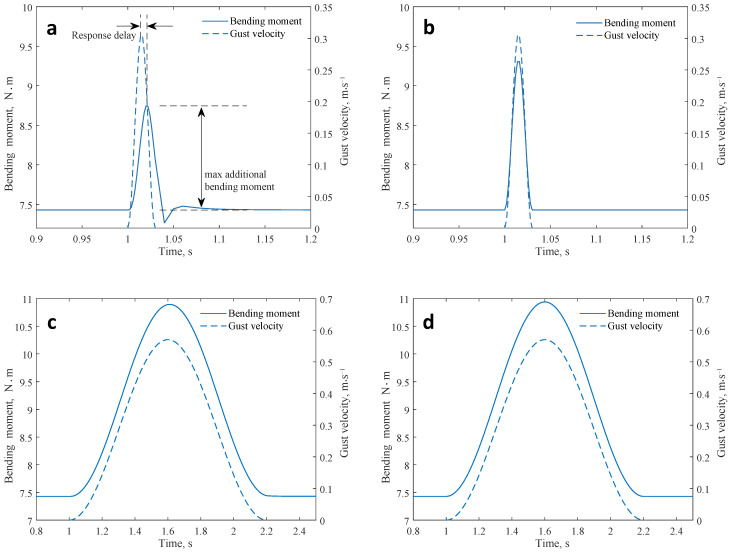
Comparison of the dynamic and quasi-static models. (**a**) λ=2c, dynamic model. (**b**) λ=2c, quasi-static model. (**c**) λ=80c, dynamic model. (**d**) λ=80c, quasi-static model.

The maximum additional bending moment increases with the gust scale, mainly due to the change in transient downwash velocity and the equivalent angle of attack. In addition, if the wing root bending moment and the gust velocity at the leading edge are plotted on the same time axis, dynamic characteristics, including response delay, can be observed. In the case of a small-scale gust, there is also an overshooting phenomenon, i.e., the minimum value of the bending moment after the gust disturbance is smaller than that before the disturbance. A comparison of bending moment simulation results of dynamic (method in this paper) and quasi-static (steady panel method + finite element method) models is given in Figure 16.

From the comparison, it can be seen that the quasi-static model predicts the maximum bending moment to a larger extent and fails to capture the phenomena of response delay and overshooting. This is mainly affected by the inertia and damping. However, as the gust scale increases, the changing rate of gust velocity decreases, making the dynamic properties of the response curve to become weaker. Thus, the results reported by the dynamic and quasi-static models converge in the case of large-scale gust.

**Figure 17:**
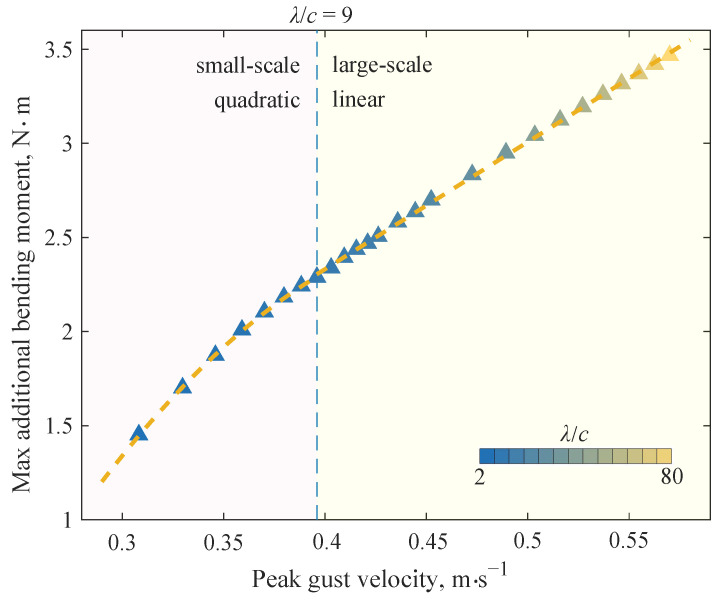
Maximum additional bending moment at the wing root.

If the variation of the maximum additional bending moment with respect to the peak gust velocity is plotted as a curve, as shown in Figure 17, a two-stage variation can be observed: the quadratic relationship in the small-scale gusts and the linear relationship in the large-scale gusts. The cut-off point occurs in the case of the gust with the scale λ=9c. The data fitting result within each part is given in Table 3, where $R^{2}$ is the coefficient of determination. It should be emphasized that the quadratic function curve does not pass through the origin point, which means that the curve cannot extrapolate the situation of gusts of scales less than 2c. The DVA is no longer applicable in such conditions.

These discoveries may help to guide the selection of the suitable computational model to simulate gust response, and will help to design the gust alleviation controller. In the following section, we describe how to realize gust alleviation using a simple controller of in-plane folding morphing of the wing.

### 4.3. Gust Alleviation

In general, the gust response of any UAV includes the attitude change in the airframe. Therefore, the actual gust alleviation should take into account the attitude change. However, in this paper, in order to show the modeling and the controller design method, a root-fixed wing is still used as an example. This simplification will not affect the obtaining of the main conclusions as well as the validity of the computational methodology.

#### 4.3.1. Wing Aeroservoelastic System

**Figure 18:**
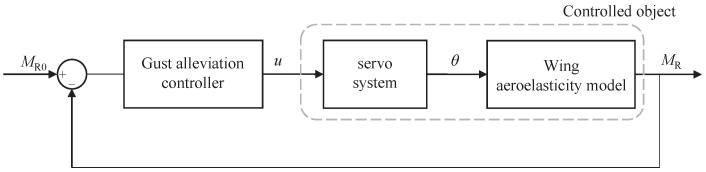
Wing aeroservoelastic system schematic.

When a controller is applied to control the wing folding angle, the wing-flow-servo actually constitutes a structure-fluid-controlling coupled aeroservoelastic (ASE) system, as shown in Figure 18.

**Figure 19:**
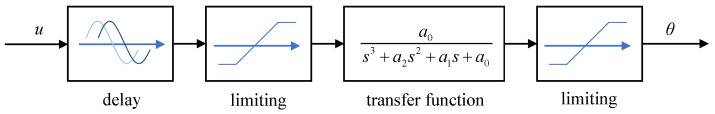
Servo system schematic.

Since the aeroelasticity model has been discussed in the previous sections, the main focus here is on the controlling issues. The servo system in the ASE system can be expanded as a third-order transfer function, a signal delay unit, and signal limiting units, as Figure 19. The first limiting unit represents the amplitude of the input signal, while the second limiting unit represents the amplitude limited by the mechanical limiter.

All the parameters in the servo system model could be tested in ground tests. Here we use a typical result: signal delay τ=0.002 s, signal limiting $\theta \in [0^{\circ },40^{\circ }]$, servo parameters $a_{0}=3.386\times 10^{6}$, $a_{1}=4.422\times 10^{4}$, $a_{2}=343.1$. Furthermore, since the transfer function is not a time-domain model and all computational methods in this paper are time-domain advancement methods, the transfer function is converted to an equivalent state–space realization. The state–space model is discreted through the zero-order holding method [32]. See Appendix B for more details on state–space modeling and discretization.

#### 4.3.2. Gust Alleviation Controller

**Figure 20:**
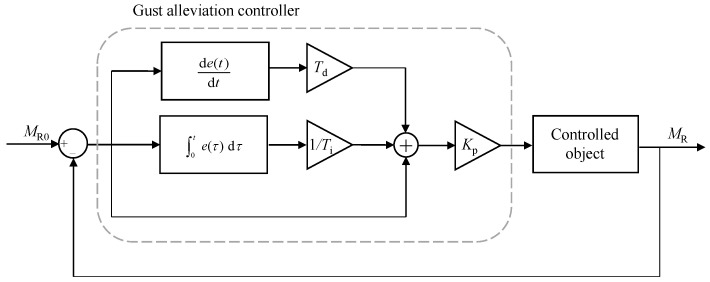
Gust alleviation controller schematic.

A classical PID controller is used to control the folding of the wing’s outer section to achieve gust load alleviation. And the control objective is to make the bending moment at the wing root $M_{R}$ close to that before disturbance $M_{R0}$, as shown in Figure 20.

**Figure 21:**
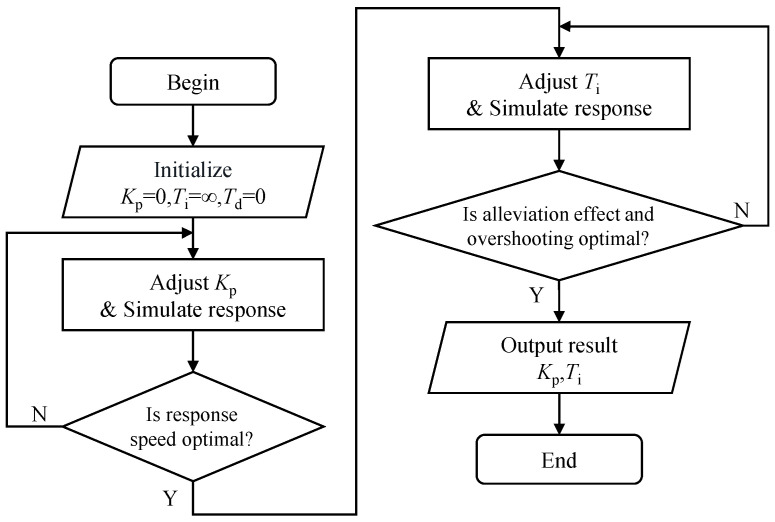
Designing of gust alleviation controller.

This controller is modeled using an incremental PID algorithm. In the design of the controller, first, the integral coefficient $T_{i}$ is set to infinity, the differential coefficient $T_{d}$ is set to 0, and the amplification factor $K_{p}$ is adjusted until the response speed of bending moment in the closed-loop system come into a suitable state. Then, the integral coefficient $T_{i}$ is adjusted until the bending moment alleviation rate and overshooting value of the closed-loop system come into a suitable state, as shown in Figure 21. Finally, the differential coefficient $T_{d}$ is adjusted to reduce the overshooting value in the closed-loop system (This step is not used in this example.)

The coefficients used in the gust alleviation controller is

(26) $$\left\{\begin{array}{cc} K_{p} & =8,\\ K_{i} & =\frac{K_{p}}{T_{i}}=280,\\ K_{d} & =K_{p}T_{d}=0. \end{array}\right.$$

Usually, the differential unit reduces the overshooting value and simultaneously reduces the response speed of the system. In addition, due to the influence of signal noise, there could be a large error in the differential calculation of the actual feedback signal, adversely affecting the control quality. Thus, we have set the differential coefficient $T_{d}$ to 0.

#### 4.3.3. Numerical Simulation

**Figure 22:**
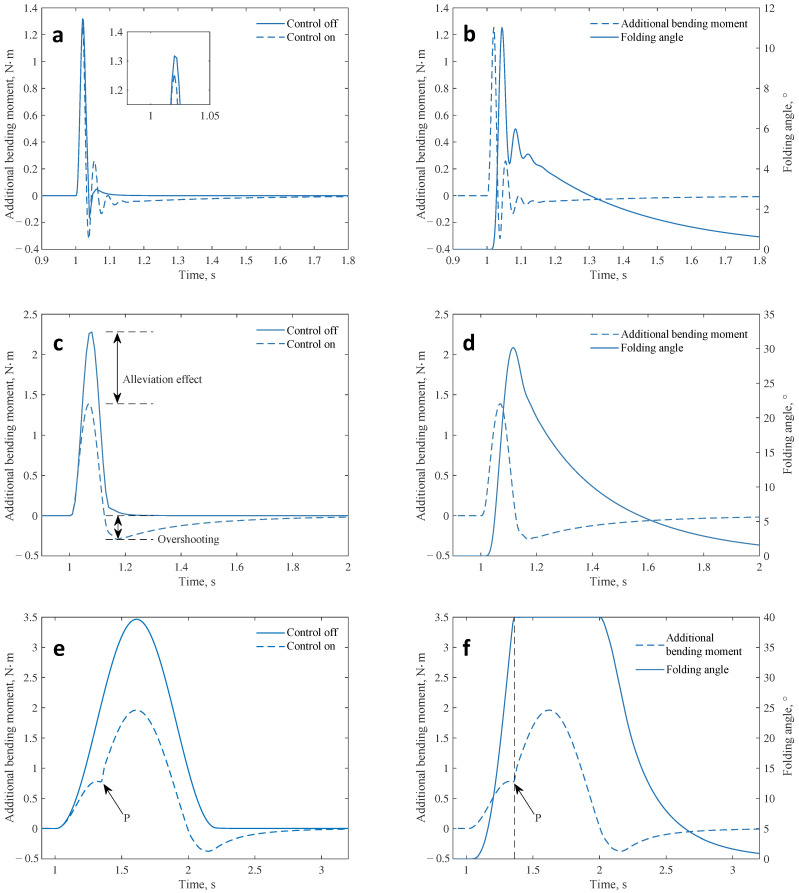
Bending moment response curve of the closed-loop system. (**a**) λ=2c, comparison of additional bending moments. (**b**) λ=2c, variation of folding angle. (**c**) λ=9c, comparison of additional bending moments. (**d**) λ=9c, variation of folding angle. (**e**) λ=80c, comparison of additional bending moments. (**f**) λ=80c, variation of folding angle.

In the numerical simulation, the system inputs are maintained at the pre-disturbance bending moment value $M_{R0}$. The gust alleviation controller then gives the control command *u* based on the current bending moment $M_{R}$ and the control target $M_{R0}$. The control command *u* is sent to the servo system as shown in Figure 19, resulting in the folding angle θ of the outer wing segment. The wing aeroelasticity model will continue to update the aerodynamic loads on the wing and give a new bending moment. These steps are repeated to obtain the bending moment response curve when the gust alleviation controller is enabled (closed-loop system), as shown in Figure 22.

The alleviation effect of the controller is affected by gust scales. Under the large-scale gust (λ=80c, Figure 22e), the alleviation effect is rewarding (43.4%), while it is unsatisfactory (5.5%) under the small-scale gust (λ=2c, Figure 22a). The important reason is the signal delay in the controller. When the gust is of a small scale (e.g., λ=2c), it takes a short time for the wing to cross the gust area. Thus, the signal delay can create a large phase difference between input and output signals in the controller, as shown in Figure 22b. In the front part, where the bending moment grows rapidly, the outer section of the wing does not receive any effective control command from the controller, so the additional bending moment is not effectively controlled. This problem will be mitigated as the gust scale increases. The additional bending moment can still be effectively controlled under the gust of a large scale. This feature likewise signifies that the dynamic model will play a critical role in high-frequency gust simulations.

Furthermore, the oscillation, the overshooting, and the inflection point are observed in the response of the closed-loop system disturbed by gusts of three typical scales, respectively. The oscillations under small-scale gusts arise mainly from the action of the proportional term. Since the gusts act for a short time, the time integral of the additional bending moment is also small. So, the control signal will be determined mainly by the proportionality coefficient $K_{p}$ and the control error $e=M_{R}-M_{R0}$. At this point, the controller exhibits the oscillating effect as proportional controllers. As the gust scale and action time increase, the above oscillations are gradually suppressed, and the closed-loop response exhibits more characteristics of an integral controller, such as overshooting (Figure 22c). As the gust scale continues to increase, the actuator reaches saturation, and the inflection phenomenon shown at point P in Figure 22e occurs.

## 5. Discussion

According to the above numerical simulation, it can be observed that the effect of the gust alleviation controller presents different characteristics depending on the gust scale, including alleviation effect and response stability. We have collated three metrics to show the effect of the controller under different scales of gusts:

- alleviation effect, the ability of the controller to suppress additional gust bending moments.
- overshooting, correlating with response stability.
- restore stability time, the time taken from the end of gust action to the moment when the bending moment comes back to the corridor of $M_{R0}\pm 2\%$.

**Figure 23:**
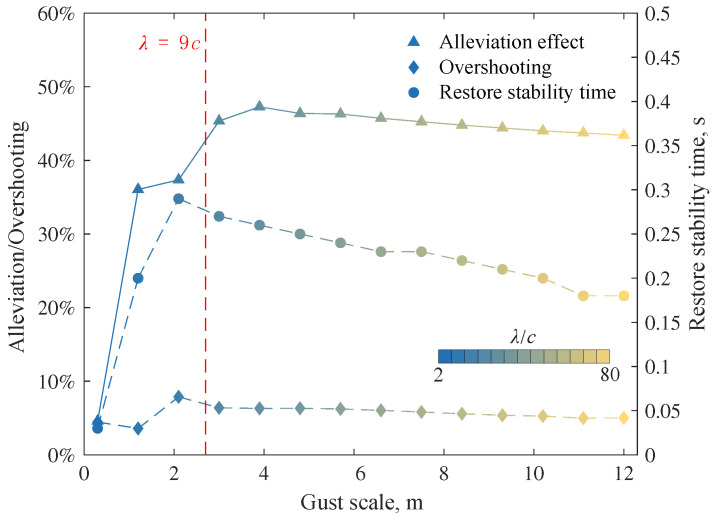
Effect of the controller under different scales of gusts.

The variation of these metrics with respect to the gust scale is shown in Figure 23.

It can be seen that the change rate of each metric with gust scale is also roughly bounded by λ=9c, which is similar to the demarcation line in Figure 17. Under the action of small-scale gusts (λ≤9c), the alleviation effect in bending moments has developed a significant change with the gust scale. The reason is mainly the signal delay and the phase difference in signals. In contrast, the gust alleviation effect tends to stabilize under large-scale gusts (λ≥9c) action. The negative effect of the delay time on the control effectiveness mainly comes from the phase difference between the folding angle and the gust load. Since the delay is almost independent of the gust scale, if the gust scale is very small, the delay time will account for a large proportion of the gust action time. It will lead to a significant phase difference between the folding angle and the gust load, making the wing folding unable to produce the expected control effect. Taking into account the delay in controller design or using a servo with lower delay may help mitigate the “scale effect” of small-scale or high-frequency gusts.

In addition, both the overshooting and the restore stability time of the system achieve their maximum values at a gust scale slightly less than 9c. The excitation frequency of the gust of 9c is

(27) $$f_{G}=\frac{u_{\infty }}{\lambda }=7.4\mathrm{Hz}.$$

The first-order modal frequency of the wing is 7.5 Hz. Therefore, when the gust is of the scale λ slightly smaller than 9c, its excitation frequency will be close to the first-order modal frequency of the wing, allowing the system to obtain great excitation and reducing the stability. However, since this gust acts for only one cycle and the wing will change its modal frequencies during folding, the excitation will not lead to resonance or divergence.

Overall, the in-plane folding wing introduces a novel mechanism of load control and can achieve effective lift control. When the gust alleviation controller is activated, it effectively mitigates the wing root bending moment of the wing under the gust excitation, which can improve structural safety and prolong the structural life. The specific effect of the gust load alleviation control is related to both the dynamic characteristics of the control system and the structural characteristics of the wing.

In order to improve the effectiveness of the control, it is necessary to link the in-plane folding mechanism with other control surfaces related to flight attitude, such as the empennage. The control of flight attitude and altitude needs to be included in future research because the gust response of any actual UAV involves simultaneous changes in these metrics, and they may have further coupled effects on the wing load.

## 6. Conclusions

This article reports an in-plane folding wing design concept inspired by bird wings. The design sensitivity is represented analytically, and the adaptive gradient optimization algorithm has been used to optimize the main beam based on cellular structures. A wing prototype with a 976 mm span is produced, and its span can be reduced to 739 mm when the wing is fully folded. The main parts of the wing are produced using 3D printing, and a small number of parts are produced using conventional cutting.

In order to solve the simulation of gust response of avian-inspired in-plane folding wings, we develop a structural modeling method applicable to the structures of time-varying parameters and establish an analytical model of aerodynamic loads by unsteady panel/viscous vortex particle hybrid method. Based on the above models, a fluid-structure coupling analysis method for folding wings is given. A wind-tunnel test is applied to verify the validity of our method for predicting wing loads. The maximum errors of wing loads in steady and unsteady cases are 2.0% and 5.8%, respectively. Then, the gust response characteristics of the wing without control are investigated.

A two-stage variation can be observed in the gust response of the wing without control, where the maximum additional wing root bending moments exhibit the quadratic and linear relationship with respect to the peak gust velocities, respectively. The cut-off point is of gust scale λ=9c. Under the action of small-scale gusts, the structural elastic deformation cannot be fully developed due to the structural inertia and system damping. So, the actual structural internal force is smaller than the results of the quasi-static model.

The incremental PID algorithm is used to design a gust alleviation controller, achieving an alleviation effect of additional wing root moments of 5.5%∼47.3% under different scales gusts action. Under the action of small-scale gusts, the signal delay in the controller results in a phase difference between the input and output signals, reducing the alleviation effect of the controller. The worst stability is achieved in cases where the gust scale is slightly less than 9c. This is due to the proximity of the gust excitation frequency to the structure’s first-order modal frequency.

The avian-inspired in-plane folding wing in this article introduces a novel mechanism of load control, and can achieve effective lift control or gust load alleviation. The results obtained may provide a reference for further research on similar bionic or large-scale morphing wings.

## Figures and Tables

**Table 1:** Process and material programs of the prototype.

Part	Process	Material	Elasticity Modulus	Density
Beam	L-PBF ^1^	AlSi10Mg	50,000 MPa	2.7 g·cm${\,}^{-3}$
Rib	L-PBF	AlSi10Mg	50,000 MPa	2.7 g·cm${\,}^{-3}$
Foldable segment	SLS ^2^	Fiber-added nylon	3500 MPa	1.1 g·cm${\,}^{-3}$
Rachis	Cutting	Aluminum alloy	70,000 MPa	2.7 g·cm${\,}^{-3}$
Skin	Laser cut	Glass fiberboard	25,000 MPa	1.8 g·cm${\,}^{-3}$

^1^ Laser powder bed fusion. ^2^ Selective laser sintering.

**Table 2:** Modal damping ratio of each mode measured at 40${\,}^{\circ }$ folding angle.

Mode No.	#1	#2	#3	#4	#5	#6
Modal damping ratio	0.0263	0.0825	0.08	0.0229	0.0402	0.0331

**Table 3:** Result of fitting the maximum additional bending moment as a function of peak gust velocity.

Gust Scale	Fitting Results *	$R^{2}$
2≤λ/c≤9	$\Delta M_{R,\max}=-35.5u_{\mathrm{ds}}^{2}+34.63u_{\mathrm{ds}}-5.855$	0.9998
λ/c≥9	$\Delta M_{R,\max}=6.76u_{\mathrm{ds}}-0.3729$	0.9994

* $\Delta M_{R,\max}$: the maximum additional bending moment at the wing root [N·m]; $u_{\mathrm{ds}}$: the peak gust velocity [m·s${\,}^{-1}$].

## Data Availability

All the necessary data have been shown in the article (or the Appendix A and Appendix B).

## Appendix A. Coefficient Matrices of the Recursive Formula

The coefficient matrices of the recursive Formula (15) are listed as follows:

(A1) $$\begin{array}{cc} H_{f}= & \frac{{\left(\Delta t\right)}^{2}}{30}\left[\begin{array}{cccc} 36 & 3 & -36 & 3\\ -3 & 1 & 33 & -4 \end{array}\right]⊗I, \end{array}$$

(A2) $$\begin{array}{cc} H_{0}= & \left[\begin{array}{cccc} 6 & 6 & 4 & 2\\ -2 & -4 & -1 & -1 \end{array}\right]\times M_{k}+\frac{\Delta t}{30}\left[\begin{array}{cccc} 18 & -18 & -21 & -9\\ 6 & 24 & 8 & 7 \end{array}\right]\times {\bar{C}}_{k}\\ \; & +\frac{{\left(\Delta t\right)}^{2}}{30}\left[\begin{array}{cccc} -33 & -3 & -3 & 0\\ 6 & -3 & 0 & -1 \end{array}\right]\times K_{k}, \end{array}$$

(A3) $$\begin{array}{cc} H_{1}= & \left[\begin{array}{cccc} 6 & 6 & -2 & -4\\ -2 & -4 & 1 & 3 \end{array}\right]\times M_{k}+\frac{\Delta t}{30}\left[\begin{array}{cccc} 18 & -18 & -9 & -21\\ 6 & 24 & 2 & 13 \end{array}\right]\times {\bar{C}}_{k}\\ \; & +\frac{{\left(\Delta t\right)}^{2}}{30}\left[\begin{array}{cccc} -3 & -33 & 0 & 3\\ 6 & 27 & -1 & -3 \end{array}\right]\times K_{k}, \end{array}$$

where the symbols × and ⊗ represent the product of compound matrices and the Kronecker product of matrices, respectively. In Equations (A2) and (A3), the mass matrix M, damping matrix $\bar{C}$, and stiffness matrix K have been multiplied by row compound matrices which consist of 2×2 2-dimensional row vectors. This structure has been marked in Equations (A2) and (A3) by dotted lines.

It should be noted that the specific values of the above matrices are not limited to the examples listed here. Depending on the treatment of the variational terms, other values may be taken [16], but they will not significantly affect the results of Equation (15).

## Appendix B. State–Space Realization of Transfer Function and Its Discretization

For the third-order transfer function studied in Figure 19, its input and output signals satisfy

(A4) $$\left(s^{3}+a_{2}s^{2}+a_{1}s+a_{0}\right)Y\left(s\right)=a_{0}U\left(s\right),$$

where Y(s) and U(s) are the input and output signals, respectively. Thus, the signals in the time domain will satisfy

(A5) $$\dddot{y}\left(t\right)+a_{2}\ddot{y}\left(t\right)+a_{1}\dot{y}\left(t\right)+a_{0}y\left(t\right)=a_{0}u\left(t\right).$$

Introduce a state quantity $x={\left[x_{1},x_{2},x_{3}\right]}^{T}={\left[y,\dot{y},\ddot{y}\right]}^{T}$, then the state–space model of the transfer function is

(A6) $$\left\{\begin{array}{cc} \dot{x} & =\mathrm{Ax}+Bu,\\ y & =\mathrm{Cx}, \end{array}\right.$$

where

(A7) $$\left\{\begin{array}{cc} A & =\left[\begin{array}{ccc} 0 & 1 & 0\\ 0 & 0 & 1\\ -a_{0} & -a_{1} & -a_{2} \end{array}\right],\\ B & ={\left[\;0\;0\;a_{0}\;\right]}^{T},\\ C & =\left[\;1\;0\;0\;\right]. \end{array}\right.$$

Apply a time discretization of the state–space model (A6), then a discrete-time state–space model can be obtained:

(A8) $$\left\{\begin{array}{cc} x_{k+1} & ={\Phi x}_{k}+Hu_{k},\\ y_{k} & ={\mathrm{Cx}}_{k}, \end{array}\right.$$

where the subscript *k* represents the number of time steps. According to zero-order holding method, recursive coefficient matrices Φ and H are both the matrix functions of the time step *T*:

(A9) $$\left\{\begin{array}{cc} \Phi (T) & =e^{AT}=L^{-1}\left(sI-A\right),\\ H(T) & =\int_{0}^{T}e^{A\tau }d\tau B, \end{array}\right.$$

where $L^{-1}$ is the inverse Laplace transform.
